# Evolution of scientific productivity in dentistry: tracking postgraduate publications at a Peruvian university

**DOI:** 10.3389/froh.2024.1494435

**Published:** 2024-12-05

**Authors:** Cesar D. Rojas-Senador, María Claudia Garcés-Elías, Roberto A. León-Manco

**Affiliations:** Facultad de Estomatología, Universidad Peruana Cayetano Heredia, Lima, Peru

**Keywords:** education, dental, graduate, students, academic dissertation, universities

## Abstract

**Introduction:**

Scientific production indicators help universities quantitatively and qualitatively assess postgraduate students’ publications and contribute to the optimization of research processes. This study aimed to determine the frequency of publication in scientific journals of the documents submitted by postgraduate dentistry students to obtain a second professional specialty title and master's and doctoral degrees at a private Peruvian university over seven years.

**Materials and methods:**

A cross-sectional study was conducted, considering the population and the records of documents submitted to obtain the second professional specialty title and the master's and doctoral degrees in dentistry at a private Peruvian university, published in its institutional repository from 2017 to 2023. The primary outcome was publication in scientific journals, and the covariates were the year of formal submission of the document, postgraduate level, modality of document preparation, area of dentistry specialty according to the American Dental Association, year of publication, international indexing, impact factor (IF), and quartile. A descriptive analysis was performed to obtain absolute and relative frequencies.

**Results:**

Between 2017 and 2023, out of 322 documents submitted to receive the second professional specialty title and the master's and doctoral degrees published in the institutional repository, 72 were published in scientific journals. Of these, 81.94% (*n* = 59) were published in an internationally indexed scientific journal, with 13.56% (*n* = 8) published in a journal with an IF≥2 and 10.18% (*n* = 6) in a Q1 category journal.

**Discussion:**

In low-income countries, the nexus between education and scientific output is multifaceted. While education serves as a critical catalyst, numerous challenges constrain the expansion of research. These nations contribute a mere 2% of global scientific production, despite confronting significant public health issues, underscoring a notable discrepancy between educational attainment and research productivity. The disparities in scientific output across universities in Latin America, Asia, and Europe are shaped by a confluence of cultural and economic determinants. In conclusion, 22.36% of the documents submitted to obtain the second professional specialty title and the master's and doctoral degrees by postgraduate dentistry students at a private Peruvian university over seven years were published in scientific journals.

## 1 Introduction

In today's society, science and technology hold significant relevance, underscoring the need for young researchers to generate and share knowledge through publications (1). Scientific journals are essential for this dissemination, and the lack of publication weakens the connection between researchers and the scientific community, hindering the application and expansion of new knowledge in health. In dentistry, publications are crucial due to the rapid evolution of techniques and treatments and their impact on public health, demonstrating students’ ability to communicate new knowledge (2). According to the American Dental Association (ADA), a specialty in dentistry is a discipline with a unique and well-defined focus based on advanced knowledge and formal skills, intending to protect patients and improve the quality of oral health care through postgraduate training (3). Similarly, master's programs in health sciences provide comprehensive professional development, focusing on technical-scientific training and the ability for independent research (4). Doctoral programs in health sciences include data management, literature critique, real-world problem-solving, communication skills development, and active participation in research (5, 6). In dentistry, these programs aim to develop critical and autonomous research competencies, preparing future educators to foster research in oral and maxillofacial health (4).

Writing and publishing a research paper in graduate school encourages inquiry and exposes students to various research techniques. These works should be disseminated within the scientific community through publications in peer-reviewed journals, indicating their scientific value and acceptance (7). Publishing in scientific journals enhances students’ professional quality and personal satisfaction, establishing them as experts and contributing to dental knowledge (8). Scientific publication is both an academic requirement and a professional opportunity for graduate students. However, the frequency and quality of these publications depend on institutional support, available resources, and the research culture of the institution (9). Measuring patterns in written scientific communication evaluates variables such as the number of publications and citations of authors, research groups, or institutions (10). In health sciences, especially dentistry, various methods are used to quantify scientific production, including impact factor, quartile, and the H-index (10, 11). These indicators have shown a gradual increase in scientific output in health in recent years, standing out as an essential factor for universities (12). Globally, dentistry has experienced notable growth in scientific production, with an increase observed between 1986 and 2008 in Web of Science and between 2009 and 2014 in PubMed, although with a reversal between 2014 and 2019 (11, 13). Dentistry faces low scientific production in Latin America in universities due to a deficient research culture (14). In Peru, scientific production in dentistry increased between 2014 and 2019 in Scopus, but with limited contributions from students, more so in private universities (15).

Evaluating scientific production using specific techniques and indicators is crucial, as it facilitates the implementation of strategies to improve and plan programs that promote research, allocate funds efficiently, and optimize resources for the benefit of the scientific community (9, 15). These indicators help universities quantitatively and qualitatively assess postgraduate students’ publications, contributing to stimulating or modifying research processes within faculties (9). Thus, this study aimed to determine the frequency of publication in scientific journals of the documents submitted by postgraduate dentistry students to obtain the second professional specialty title and the master's and doctoral degrees at a private Peruvian university over seven years.

## 2 Materials and methods

The present study adopted a cross-sectional design, covering all records of documents submitted to obtain the second professional specialty title and the master's and doctoral degrees in the programs offered by the Faculty of Stomatology at the Universidad Peruana Cayetano Heredia (UPCH) in Lima, Peru, published in the institutional repository between 2017 and 2023. The entire population was analyzed, and no records were excluded, as all documents contained the complete information required to determine their publication status in a scientific journal.

In this study, the primary outcome was publication in scientific journals. The covariates included the year of formal submission of the document, the postgraduate level, the modality of document preparation, the area of dental specialty according to the American Dental Association (ADA), the year of publication, international indexing, impact factor, and quartile.

The publication in scientific journals was qualitatively categorized as “No” if the document was not published in a scientific journal and “Yes” if it was published. The postgraduate level, another qualitative variable, was classified into “Second Professional Specialty,” “Master's,” and “Doctorate.” The modality of document preparation was divided into “Individual” for documents prepared by a single student and “Collective” for those prepared by two or more students. The area of dental specialty, according to the ADA, was classified into nine categories: “Oral and Maxillofacial Surgery,” “Endodontics,” “Pediatric Dentistry,” “Orthodontics and Dentofacial Orthopedics,” “Oral and Maxillofacial Pathology,” “Periodontics,” “Prosthodontics and Restorative Dentistry,” “Oral and Maxillofacial Radiology,” and “Dental Public Health,” specialties recognized by the National Commission on Recognition of Dental Specialties and Certifying Boards (NCRDSCB) according to ADA requirements (16).

International indexing, as a qualitative variable, was classified into two categories: “No” if the document was published in a journal not indexed in Scopus, Web of Science, or SciELO, and “Yes” if the publication was made in a journal indexed in any of these databases. The journal's impact factor (IF), representing the average number of citations received per article published in that journal over the previous two years (17), was categorized into “IF < 2” for journals with an impact factor less than 2 and “IF ≥ 2” for those with an impact factor of 2 or higher. The quartile, another qualitative variable, is used to measure the relevance of a journal within its field based on the distribution of impact factors, journals are ranked into quartiles by dividing them into four equal groups according to their impact factor within a specific subject area; the top 25% of journals are classified as “Q1”, the next 25% as “Q2” (25th to 50th percentiles), followed by “Q3” (50th to 75th percentiles), and the bottom 25% as “Q4”, if a journal did not have a recorded impact factor, it was categorized as “Not recorded”, this classification was derived from sources like the Journal Citation Reports (JCR) and Scimago Journal Rank (SJR).

The data on the year of submission, postgraduate level, preparation modality, and area of specialty according to the ADA were collected from the institutional repository of UPCH, a digital portal managed by the University Directorate for the Promotion and Management of Research, Science, and Technology. Information on publication in scientific journals, year of publication, international indexing, impact factor, and quartile was obtained from various databases and search engines (Scopus, Web of Science, SciELO, PubMed, and Google Scholar) through a systematized search conducted by the researchers. A document was considered published if the authors, title, and abstract matched the information of the article found.

The statistical software STATA v. 18.0 was used to perform the descriptive analysis of the variables and obtain absolute and relative frequencies. Additionally, Microsoft Excel was used to organize and present the results in tables and figures. The University Directorate of Regulatory Affairs for Research approved the study protocol at UPCH (ethical file CAR-DUARI-213-23, approved on August 2, 2023).

## 3 Results

Between 2017 and 2023, 322 documents were published in the institutional repository of UPCH to obtain the second professional specialty title and the master's and doctoral degrees in the programs offered by the Faculty of Stomatology. Of these, 22.36% (*n* = 72) were subsequently published in a scientific journal. Analyzing the year of formal submission of the documents, the highest number of publications originated from documents submitted in 2018 (*n* = 16), representing 31.37% of the documents submitted that year. In contrast, the lowest number of publications came from documents submitted in 2023 (*n* = 2), corresponding to 3.08% of the documents for that year. Regarding the postgraduate level, students in second professional specialty programs generated the highest number of publications (*n* = 58), representing 28.02% of their documents. Doctoral students had the lowest number of publications (*n* = 5), equivalent to 35.71% of their documents. Concerning the modality of preparation, most of the documents published in scientific journals were individually prepared (*n* = 56), representing 20.66% of the individual documents. Collectively prepared and published documents totaled 16, representing 31.37% of the collaborative documents. Finally, considering the area of dental specialty according to the ADA, it was found that most published documents addressed topics in Oral and Maxillofacial Radiology (*n* = 21), representing 30.43% of the documents in this specialty. In contrast, the lowest number of publications was recorded in the areas of Oral and Maxillofacial Surgery (*n* = 1) and Oral and Maxillofacial Pathology (*n* = 1), corresponding to 8.33% and 14.29% of their respective documents (Table 1).

**Table 1:** Evolution of scientific productivity in dentistry: tracking postgraduate publications at a Peruvian university, 2017–2023.

Variables	*n*	%	Publication in scientific journals				Year of publication															
			No		Yes		2017		2018		2019		2020		2021		2022		2023		2024	
			*n*	%	*n*	%	*n*	%	*n*	%	*n*	%	*n*	%	*n*	%	*n*	%	*n*	%	*n*	%
Total	322	100.00	250	77.64	72	22.36	3	4.17	2	2.78	9	12.50	15	20.83	14	19.44	17	23.61	10	13.89	2	2.78
Year of formal document submission																						
2017	38	11.80	29	76.32	9	23.68	2	22.22	1	11.11	1	11.11	1	11.11	1	11.11	2	22.22	1	11.11	0	0.00
2018	51	15.84	35	68.63	16	31.37	0	0.00	1	6.25	6	37.50	6	37.50	3	18.75	0	0.00	0	0.00	0	0.00
2019	50	15.53	38	76.00	12	24.00	1	8.33	0	0.00	2	16.67	5	41.67	3	25.00	0	0.00	1	8.33	0	0.00
2020	48	14.90	37	77.08	11	22.92	0	0.00	0	0.00	0	0.00	3	27.27	4	36.37	3	27.27	1	9.09	0	0.00
2021	40	12.42	25	62.50	15	37.50	0	0.00	0	0.00	0	0.00	0	0.00	2	13.33	10	66.67	3	20.00	0	0.00
2022	30	9.32	23	76.67	7	23.33	0	0.00	0	0.00	0	0.00	0	0.00	1	14.29	2	28.57	4	57.14	0	0.00
2023	65	20.19	63	96.92	2	3.08	0	0.00	0	0.00	0	0.00	0	0.00	0	0.00	0	0.00	0	0.00	2	100.00
Postgraduate level																						
Second professional speciality	207	64.28	149	71.98	58	28.02	3	5.17	2	3.45	8	13.79	14	24.14	9	15.52	12	20.69	8	13.79	2	3.45
Master's	101	31.37	92	91.09	9	8.91	0	0.00	0	0.00	1	11.11	1	11.11	4	44.45	2	22.22	1	11.11	0	0.00
Doctorate	14	4.35	9	64.29	5	35.71	0	0.00	0	0.00	0	0.00	0	0.00	1	20.00	3	60.00	1	20.00	0	0.00
Modality of document preparation																						
Individual	271	84.16	215	79.34	56	20.66	3	5.36	1	1.79	7	12.50	11	19.64	12	21.43	13	23.21	7	12.50	2	3.57
Collective	51	15.84	35	68.63	16	31.37	0	0.00	1	6.25	2	12.50	4	25.00	2	12.50	4	25.00	3	18.75	0	0.00
Area of dental specialty according to the ADA																						
Oral and maxillofacial surgery	12	3.73	11	91.67	1	8.33	0	0.00	0	0.00	0	0.00	0	0.00	0	0.00	1	100.00	0	0.00	0	0.00
Endodontics	27	8.39	22	81.48	5	18.52	0	0.00	0	0.00	1	20.00	0	0.00	2	40.00	0	0.00	1	20.00	1	20.00
Pediatric dentistry	26	8.07	16	61.54	10	38.46	0	0.00	1	10.00	0	0.00	3	30.00	2	20.00	2	20.00	2	20.00	0	0.00
Orthodontics and dentofacial orthopedics	46	14.29	38	82.61	8	17.39	0	0.00	0	0.00	0	0.00	2	25.00	3	37.50	2	25.00	1	12.50	0	0.00
Oral and maxillofacial pathology	7	2.17	6	85.71	1	14.29	0	0.00	0	0.00	0	0.00	0	0.00	1	100.00	0	0.00	0	0.00	0	0.00
Periodontics	24	7.45	21	87.50	3	12.50	1	33.33	0	0.00	0	0.00	0	0.00	0	0.00	2	66.67	0	0.00	0	0.00
Prosthodontics and restorative dentistry	61	18.94	46	75.41	15	24.59	1	6.67	1	6.67	3	20.00	4	26.66	1	6.67	4	26.66	1	6.67	0	0.00
Oral and maxillofacial radiology	69	21.43	48	69.57	21	30.43	1	4.75	0	0.00	5	23.81	5	23.81	3	14.29	4	19.05	3	14.29	0	0.00
Dental public health	50	15.53	42	84.00	8	16.00	0	0.00	0	0.00	0	0.00	1	12.50	2	25.00	2	25.00	2	25.00	1	12.50

*n*, absolute frequency; %, relative frequency.

**Figure 1:**
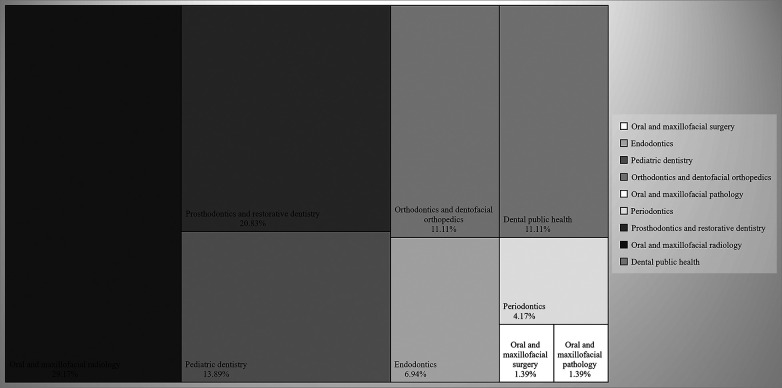
Evolution of scientific productivity in dentistry by area of dental specialty according to the ADA: tracking postgraduate publications at a Peruvian university, 2017–2023.

When graphing the distribution of all the documents (*n* = 72) for obtaining the second professional specialty title and the master's and doctoral degrees that were published in a scientific journal, categorized by the area of dental specialty as defined by the ADA, it was observed that Oral and Maxillofacial Radiology was the most addressed area, with 29.17% (*n* = 21) of the published documents. In contrast, the least addressed areas were Oral and Maxillofacial Surgery and Oral and Maxillofacial Pathology, each representing 1.39% (*n* = 1) of the published documents (Figure 1).

Between 2017 and 2024, 72 documents for obtaining the second professional specialty title and the master's and doctoral degrees were published in various scientific journals. Of these, 81.94% (*n* = 59) were published in internationally indexed scientific journals in Scopus, Web of Science, or SciELO databases. Regarding the year of formal submission, the hihest number of documents published in internationally indexed journals were submitted in 2018 (*n* = 14), representing 87.50% of the documents submitted that year. The lowest number was submitted in 2023 (*n* = 2), corresponding to 100% of the documents submitted that year. Regarding the postgraduate level, students in second professional specialty programs authored most of the documents published in internationally indexed journals (*n* = 45), representing 77.59% of their publications. Doctoral students published 5 documents in internationally indexed journals, representing 100% of their publications. Concerning the modality of preparation, most of the documents published in internationally indexed journals were prepared individually (*n* = 49), representing 87.50% of individual publications. In comparison, collectively prepared and published documents in internationally indexed journals totaled 10, representing 62.50% of collective publications. Considering the area of dental specialty according to the ADA, most of the documents published in internationally indexed journals addressed topics in Oral and Maxillofacial Radiology (*n* = 17), representing 80.95% of the publications in this area. Additionally, 100% of the documents in the areas of Oral and Maxillofacial Surgery (*n* = 1), Orthodontics and Dentofacial Orthopedics (*n* = 8), Oral and Maxillofacial Pathology (*n* = 1), Periodontics (*n* = 3), and Dental Public Health (*n* = 8) were published in internationally indexed scientific journals (Table 2).

**Table 2:** Evolution of scientific productivity in dentistry: tracking postgraduate publications at a Peruvian university (2017–2023), according to international indexing.

Variables	*n*	%	International indexing				Impact factor (IF)				Quartile									
			No		Yes		IF<2		IF≥2		Not recorded		Q1		Q2		Q3		Q4	
			*n*	%	*n*	%	*n*	%	*n*	%	*n*	%	*n*	%	*n*	%	*n*	%	*n*	%
Total	72	100.00	13	18.06	59	81.94	51	86.44	8	13.56	39	66.10	6	10.18	5	8.47	4	6.78	5	8.47
Year of formal document submission																				
2017	9	12.50	0	0.00	9	100.00	8	88.89	1	11.11	6	66.67	1	11.11	1	11.11	0	0.00	1	11.11
2018	16	22.22	2	12.50	14	87.50	13	92.86	1	7.14	9	64.28	0	0.00	2	14.29	2	14.29	1	7.14
2019	12	16.67	4	33.33	8	66.67	7	87.50	1	12.50	6	75.00	1	12.50	0	0.00	1	12.50	0	0.00
2020	11	15.28	3	27.27	8	72.73	7	87.50	1	12.50	5	62.50	1	12.50	0	0.00	0	0.00	2	25.00
2021	15	20.83	2	13.33	13	86.67	10	76.92	3	23.08	8	61.55	2	15.38	2	15.38	0	0.00	1	7.69
2022	7	9.72	2	28.57	5	71.43	4	80.00	1	20.00	3	60.00	1	20.00	0	0.00	1	20.00	0	0.00
2023	2	2.78	0	0.00	2	100.00	2	100.00	0	0.00	2	100.00	0	0.00	0	0.00	0	0.00	0	0.00
Postgraduate level																				
Second professional speciality	58	80.56	13	22.41	45	77.59	41	91.11	4	8.89	35	77.77	3	6.67	1	2.22	3	6.67	3	6.67
Master's	9	12.50	0	0.00	9	100.00	8	88.89	1	11.11	3	33.33	1	11.11	3	33.33	1	11.11	1	11.11
Doctorate	5	6.94	0	0.00	5	100.00	2	40.00	3	60.00	1	20.00	2	40.00	1	20.00	0	0.00	1	20.00
Modality of document preparation																				
Individual	56	77.78	7	12.50	49	87.50	42	85.71	7	14.29	30	61.23	5	10.20	5	10.20	4	8.17	5	10.20
Collective	16	22.22	6	37.50	10	62.50	9	90.00	1	10.00	9	90.00	1	10.00	0	0.00	0	0.00	0	0.00
Area of dental specialty according to the ADA																				
Oral and maxillofacial surgery	1	1.39	0	0.00	1	100.00	1	100.00	0	0.00	1	100.00	0	0.00	0	0.00	0	0.00	0	0.00
Endodontics	5	6.94	1	20.00	4	80.00	4	100.00	0	0.00	2	50.00	0	0.00	0	0.00	1	25.00	1	25.00
Pediatric dentistry	10	13.89	7	70.00	3	30.00	2	66.67	1	33.33	1	33.33	1	33.33	1	33.33	0	0.00	0	0.00
Orthodontics and dentofacial orthopedics	8	11.11	0	0.00	8	100.00	5	62.50	3	37.50	3	37.50	2	25.00	1	12.50	1	12.50	1	12.50
Oral and maxillofacial pathology	1	1.39	0	0.00	1	100.00	0	0.00	1	100.00	0	0.00	1	100.00	0	0.00	0	0.00	0	0.00
Periodontics	3	4.17	0	0.00	3	100.00	1	33.33	2	66.67	1	33.33	1	33.33	1	33.33	0	0.00	0	0.00
Prosthodontics and restorative dentistry	15	20.83	1	6.67	14	93.33	13	92.86	1	7.14	12	85.72	1	7.14	0	0.00	0	0.00	1	7.14
Oral and maxillofacial radiology	21	29.17	4	19.05	17	80.95	17	100.00	0	0.00	15	88.24	0	0.00	1	5.88	1	5.88	0	0.00
Dental public health	8	11.11	0	0.00	8	100.00	8	100.00	0	0.00	4	50.00	0	0.00	1	12.50	1	12.50	2	25.00

*n*, absolute frequency; %, relative frequency.

Regarding the impact factor (IF) and considering exclusively the documents for obtaining the second professional specialty title and the master's and doctoral degrees published in an internationally indexed scientific journal (*n* = 59), 86.44% (*n* = 51) were published in journals with an IF < 2. In comparison, 13.56% (*n* = 8) were published in journals with an IF ≥ 2. Analyzing the year of formal submission, the highest number of formally submitted documents in 2021 were published in journals with an IF ≥ 2 (*n* = 3), representing 23.08% of the papers from that year published in an internationally indexed journal. Regarding the postgraduate level, students in second professional specialty programs authored most of the documents published in journals with an IF ≥ 2 (*n* = 4), representing 8.89% of their publications in internationally indexed journals. Regarding the modality of preparation, most of the documents with an IF ≥ 2 were individually prepared (*n* = 7), representing 14.29% of individual publications in internationally indexed journals. Considering the area of dental specialty according to the ADA, the highest number of documents published in journals with an IF ≥ 2 focused on Orthodontics and Dentofacial Orthopedics (*n* = 3), representing 37.50% of the publications in this area in internationally indexed journals (Table 2).

When evaluating the quartile, it was found that of the total documents for obtaining the second professional specialty title and the master's and doctoral degrees published in internationally indexed scientific journals (*n* = 59), 66.10% (*n* = 39) were published in journals not ranked according to the impact factor value. The remaining percentage was distributed among journals in Q4 with 8.47% (*n* = 5), Q3 with 6.78% (*n* = 4), Q2 with 8.47% (*n* = 5), and Q1 with 10.18% (*n* = 6). Concerning the year of formal submission, the highest number of documents published in Q1 journals were submitted in 2021 (*n* = 2), representing 15.38% of the papers submitted that year and published in an internationally indexed journal. Considering the postgraduate level, students in second professional specialty programs authored the highest number of documents published in Q1 journals (*n* = 3), representing 6.67% of their publications in internationally indexed journals. Regarding the modality of preparation, most of the documents published in Q1 journals were individually prepared (*n* = 5), representing 10.20% of individual publications in internationally indexed journals. Concerning the area of dental specialty, according to the ADA, the highest number of documents published in Q1 journals addressed topics in Orthodontics and Dentofacial Orthopedics (*n* = 2), representing 25.00% of the publications in this area in internationally indexed journals (Table 2).

## 4 Discussion

The relationship between regional scientific output and the geographical location of research institutions is inherently complex and primarily shaped by factors such as economic development, institutional longevity, and regional demand for research. Nations with higher per capita income generally produce a greater volume of citable publications, a consequence of more substantial investments in research and development (18). Additionally, the age of universities plays a pivotal role in scientific output; older institutions tend to have higher publication rates, indicating that established academic settings promote increased research productivity (19). Interregional accessibility, often facilitated through inter-institutional agreements, fosters the exchange of knowledge, with smaller regions particularly benefiting from these collaborations (20). A further consideration is the regional demand for scientific inquiry, which shapes research output in a manner contingent on the specific industrial landscape (21).

In low-income countries, the nexus between education and scientific output is equally multifaceted. While education serves as a critical catalyst, numerous challenges constrain the expansion of research. These nations contribute a mere 2% of global scientific production, despite confronting significant public health issues, underscoring a notable discrepancy between educational attainment and research productivity (22). The disparities in scientific output across universities in Latin America, Asia, and Europe are shaped by a confluence of cultural and economic determinants. Although financial resources are indispensable, cultural perceptions of science play an equally crucial role in shaping scientific productivity. European universities, benefitting from more robust funding, exhibit higher publication and citation rates (23). Conversely, Latin American institutions face acute financial limitations that curtail their research capabilities (24). Researchers in the region frequently operate with inadequate facilities and restricted access to essential resources, impeding the advancement of high-caliber research (25). Moreover, in many Latin American countries, science is not regarded as a pivotal driver of economic development, resulting in minimal governmental support and limited public interest in scientific endeavors (24). Nonetheless, researchers trained abroad—particularly in Europe and North America—often demonstrate elevated levels of productivity, suggesting that exposure to diverse academic cultures fosters enhanced scientific output (26).

The discussion on scientific production in dentistry reveals a complex interplay of factors influencing research quality and impact globally. Bibliometric indicators, such as impact factor and quartiles, play a crucial role in evaluating and ranking institutions and countries in dental research (27). However, it is essential to acknowledge the limitations of these indicators, such as the minimal differentiation between quartile boundaries, which calls into question their effectiveness as a sole measure of quality (28). In the international context, universities such as Sichuan University and the University of Zurich stand out for their high production of highly cited articles and quality systematic reviews (29, 30). These institutions benefit from robust international collaborations and a focus on high-demand areas such as implantology and periodontics. In contrast, this study's results from a Peruvian university depict a different reality, with lower publication rates and less presence in high-impact journals. This disparity reflects the challenges faced by institutions in developing countries, including limitations in research infrastructure and funding. Globally, while countries like the United States and China lead scientific production in dentistry (30), Latin America, including Peru, shows lower citation metrics (31). This gap underscores the need for specific strategies to improve the quality and visibility of dental research in developing regions. Despite these challenges, the growing global attention to dental research offers opportunities to enhance publication practices and the quality of dental education. In this context, the findings on scientific production at a Peruvian university not only provide a baseline for future improvements but also highlight the importance of considering both local and global factors that influence productivity and impact of dental research in resource-limited settings.

The intensifying global competition in university rankings has heightened the pressure on scientific output within postgraduate programs (32). The results of this study reveal a modest increase in the number of scientific publications produced by postgraduate dental students at a private Peruvian university over the past seven years. Nevertheless, the proportion of papers published in internationally indexed journals remains low compared to the total submissions for second professional specialty titles and master's and doctoral degrees. This issue underscores a shortfall in initiatives to foster research, consequently impeding students’ capacity to critically address dental challenges (33). When these findings are contrasted with prior studies, a comparable pattern emerges across other international contexts. For instance, Obuku et al. reported limited scientific productivity among master's and doctoral students in health sciences across low- and middle-income countries in Asia, Africa, and South America (34). Likewise, Nour-Eldein et al. observed that only 21.60% of master's and doctoral theses in general and family medicine at Suez Canal University, Egypt, were published in scientific journals, with international publication rates of 71.4% and 40%, respectively, a trend that contrasts with the results of the present study (7).

Despite the rise in academic output in Peru, the rate of publication remains notably low. Mamani-Benito et al. reported that only five master's theses in health sciences were published in scientific journals between 2010 and 2019, the majority of which were not indexed internationally (8). Similarly, Fernández-Giusti et al. documented an increase in doctoral thesis defenses in health sciences between 2014 and 2019; however, by the conclusion of their study, only three theses had progressed towards publication in scientific journals (35). It is well established that higher levels of education contribute to enhanced scientific productivity, particularly in low-income regions. In Southeast Asia, master's and doctoral programs have bolstered research capacities, resulting in increased scientific output. Furthermore, the establishment of inter-institutional collaborations has significantly enhanced both publication and citation rates within the field of dentistry (36). In Latin America, countries such as Brazil, which offer greater access to postgraduate education in dentistry, yield more substantial scientific contributions. Although citation rates in the region are relatively lower, the quality of research remains comparable to that of higher-income regions, emphasizing the critical role of educational attainment (31).

Dental research varies considerably across specialties. The findings of this study reveal a marked trend of research in oral and maxillofacial radiology among postgraduate students at a Peruvian dental school. In contrast, the most researched areas in Latin America include dental public health, pediatric dentistry, and oral pathology, focusing on community health and disease management (37). A probable explanation for the discrepancies observed in the areas of dentistry that are more frequently researched could be attributed to several institutional and contextual factors (38). First, the availability of specialized faculty and research mentors in specific areas likely influences the choice of research topics by postgraduate students (39). For instance, the greater focus on Oral and Maxillofacial Radiology in this study may reflect stronger institutional expertise, better access to technology, and more structured academic programs in this field. On the other hand, areas such as Oral and Maxillofacial Surgery and Oral Pathology, which had fewer published studies, may lack sufficient institutional support, access to specialized equipment, or funding, making them less attractive or feasible for students to pursue (40). Additionally, the relevance of certain research areas to local public health needs or priorities could also influence these trends, as areas more aligned with pressing regional health issues might receive more attention from students and faculty (41).

Moreover, the institutional research agenda and the availability of external grants or funding opportunities in specific fields can further skew research focus towards more well-supported areas. Addressing these discrepancies may require a more balanced allocation of resources and mentorship across all dental specialties, fostering a more diverse range of research topics within postgraduate programs. The results suggest the need for more effective university policies to encourage students to publish their research. Strategies such as mentorship programs, scientific writing workshops, and access to dedicated research funding could significantly improve scientific productivity. These initiatives would address the limited access to high-impact journals and the persistent language barriers, which remain significant challenges in integrating Latin American research into the global scientific community (15, 31, 42).

The principal limitation of this study lies in its focus on a single private Peruvian university. Moreover, the analysis was exclusively descriptive, which offers an overview rather than a comprehensive assessment of publication quality and impact and, in turn, constrains the ability to compare results with other studies due to the limited availability of similar research in dentistry. Likewise, not directly contacting postgraduate students to confirm publication status is a potential limitation of the study's methodology. While direct contact could have provided additional insights, it might also have introduced recall bias or inconsistencies in reporting. Nonetheless, the approach used may have resulted in some underreporting if publications appeared in journals not captured by the databases consulted. Future research could benefit from more sophisticated analytical methods to provide a more accurate understanding of publication quality and impact. A mixed-method approach, combining database searches with student surveys or interviews, could provide a more comprehensive view of publication outcomes and capture any publications that may have been missed. Additionally, it would be important to investigate the factors influencing scientific publication in dental postgraduate programs at additional national universities or expand the scope to include regional and global studies.

In conclusion, the description of publication rates among postgraduate dentistry students at a private Peruvian university reveals both challenges and opportunities in the landscape of dental research in developing countries. While the overall publication rate of 22.36% indicates room for growth, it also represents a significant first step in fostering a culture of scientific inquiry and dissemination within this academic setting. The presence of publications in high-impact journals, with an impact factor of 2 or higher and classified as Q1 or Q2, albeit limited, demonstrates the potential for meaningful contributions to global dental knowledge from emerging research environments. These findings underscore the need for targeted interventions to enhance research capacity, improve writing skills, and facilitate international collaborations.

The observed publication patterns highlight the complex interplay between educational attainment and research productivity in resource-limited settings, pointing to the necessity of institutional and national policies that prioritize research infrastructure and support. Moving forward, this study serves as a crucial baseline for measuring progress and informing strategies to increase both the quantity and quality of dental research output. By addressing the identified gaps and building on existing strengths, institutions in similar contexts can work towards greater integration into the global scientific community, ultimately contributing to the advancement of dental science and practice worldwide.

## Data Availability

The original contributions presented in the study are included in the article/Supplementary Material, further inquiries can be directed to the corresponding author/s.
